# Transcriptional analysis of genes associated with glycolysis in *Streptomyces coelicolor* M145

**DOI:** 10.1007/s10123-025-00744-6

**Published:** 2025-11-04

**Authors:** Ingrid V. Alanis-Pérez, Verónica Jiménez-Jacinto, Jonathan Alanis-Péreza, María Elena Flores

**Affiliations:** 1 Departamento de Biología Molecular y Biotecnología, Instituto de Investigaciones Biomédicas, Universidad Nacional Autónoma de México, Ciudad de México, 04510 México; 2 Unidad Universitaria de Secuenciación Masiva y Bioinformática, Instituto de Biotecnología, Universidad Nacional Autónoma de México, Cuernavaca, Morelos CP 62210 México

**Keywords:** Streptomyces coelicolor, Glycolytic pathway, RNA expression, Carbon metabolism, Enzyme activity

## Abstract

We previously reported the mRNA levels of genes involved in the tricarboxylic acid cycle and the phosphoenolpyruvate-pyruvate-oxaloacetate node. Owing to the importance of the glycolytic pathway, massive mRNA sequencing of *Streptomyces coelicolor* grown in minimal medium with glucose as the sole carbon source was performed. The results revealed a large discrepancy between the mRNA levels of all the genes analysed; *sco1947*, encoding glyceraldehyde 3-phosphate dehydrogenase, had the highest relative levels with respect to those of the *hrdB* gene, with a decreasing trend over time in most cases. On the other hand, the genes encoding glucose kinase had the lowest expression levels. The existence of gene multiplicity in *Streptomyces* is well known, and *S. coelicolor* is no exception. Two, three, and even four paralogous genes were detected for some of the activities in this pathway, and not all of them were expressed under the growth conditions used. The results obtained showed the large differences in gene expression and in the activity of the enzymes involved in the glycolytic pathway, which may allow the design of strategies aimed at a better carbon flow in this microorganism.

**Supplementary Information:**

The online version contains supplementary material available at 10.1007/s10123-025-00744-6.

## Introduction

Metabolism is a very important process in all living organisms and involves a series of enzymatic reactions catalysed by proteins, which can be encoded by one or more genes (Rojas-Pirela et al. 2020). Cell growth and catabolic rates are modulated by multilevel regulatory machinery consisting of gene expression (transcriptional regulation), posttranscriptional regulation, translation, and posttranslational regulation to ultimately modulate metabolic fluxes (or enzymatic reaction rates). In addition, primary metabolites such as acetyl-CoA, glucose-6-phosphate, glyceraldehyde-3-phosphate, α-ketoglutarate and oxaloacetate serve as building blocks for secondary metabolites. Metabolites outside central carbon metabolism also contribute to the biosynthesis of secondary metabolites, such as L-valine, which is used for both methylmalonyl-CoA and ethylmalonyl-CoA biosynthesis. Furthermore, the supply of secondary metabolite precursors can be affected by variations in primary metabolite levels. Therefore, the availability of these molecules is a key factor in determining the production of secondary metabolites such as antibiotics (Romero-Rodríguez et al. 2011).

Actinomycetes, a phylum of gram-positive bacteria known for their filamentous growth pattern and high GC (guanine + cytosine) content, are prolific producers of bioactive secondary metabolites and an inexhaustible source of antibiotics (Liu et al. 2025). In this phylum, the genus *Streptomyces* stands out as a prominent source of bioactive compounds, and it generates 50–55% of the approximately 10,000 known antibiotics with diverse pharmacological activities (Subramani and Aalbersberg 2012). Secondary metabolites are nonessential for the cellular functions of *Streptomyces* but play vital roles in their ecological interactions and survival strategies. Among the bioactive compounds produced by *Streptomyces*, antibiotics have garnered significant interest (Patel et al. 2025). The biosynthesis and production of antibiotics have been widely studied; however, carbon metabolism, from which the precursors of these compounds are derived, has been neglected. High-level production of antibiotics in streptomycetes requires engineering of primary metabolism; therefore, determining the limiting steps in glucose catabolism pathways is important.

*Streptomyces coelicolor* is the best studied member of this genus and has been reported to possess the genes necessary to carry out glycolysis (van Keulen et al. 2011). Multiple gene copies encoding isoenzymes are a common feature of *S. coelicolor* and another *Streptomyces* species (Takahashi et al. 2022). This presents a challenge because it is not known whether all homologous genes are expressed under the same growth conditions. RNA sequencing provides the opportunity to determine which genes are expressed during the growth of a microorganism; thus, total RNA sequencing of *S. coelicolor* M145 grown in minimal medium with glucose as the sole carbon source was carried out to determine which of the genes of the Embden–Meyerhof–Parnas glycolytic pathway are transcribed in this microorganism.

## Materials and methods

### Culture conditions

To prepare fresh *Streptomyces coelicolor* M145 spores, mannitol-soya flour (MS) agar plates were inoculated and cultivated at 30 °C for 3 days. 1 × 10^8^ fresh spores were used to inoculate 200 mL of Luria–Bertani (LB) medium in a 1 L Erlenmeyer flask and incubated at 30 °C for 24 h with orbital shaking at 200 rpm. Mycelia from this culture were collected by centrifugation for 5 min at 4200 × *g*. The mycelium pellet was subsequently washed twice with sterile distilled water and resuspended in 5 mL of water. These mycelial suspensions were used to inoculate three Fernbach siliconized flasks containing 500 mL of minimal medium (5 g of NaCl, 2 g of (NH_4_)_2_SO_4_, 0.3 g of K_2_HPO_4_, 0.5 g of MgSO_4_.7H_2_O, 0.02 g of FeSO_4_, 0.05 g of ZnSO_4_, 0.02 g of CaCl_2_, 0.001 g of MnCl_2_, 0.001 g of CoCl_2_, 20 g of MOPS, 50 g of PEG 8000, and 10 g of glucose as a carbon source per litre) with steel coils placed at the bottom. The cultures were incubated at 30 °C with orbital shaking at 200 rpm, and each flask was sampled every 24 h for 72 h to measure growth and enzyme activity and to extract RNA.

## RNA extraction

*S. coelicolor* mycelia samples were collected and resuspended on appropriate volume of RNA Protect (Qiagen, Hilden, Germany) at each time point (24, 48 and 72 h). RNA isolation was performed using a RNeasy Mini Kit (QIAgen, Hilden, Germany) following the manufacturer’s instructions. Afterwards, the RNA was digested with a DNase Max Kit (QIAgen, Manchester, UK). The purity and integrity of the RNA were analysed by electrophoresis and in an Agilent Bioanalyzer (Agilent, Santa Clara, CA, USA). All the samples had an RNA integrity of at least 7.2, indicating that they were suitable for sequencing. The samples were used to create libraries at the Massive DNA Sequencing Unit at UNAM and were then sequenced using the Illumina NS500 platform (Illumina, San Diego, CA, USA). The study was performed using the high-performance computing cluster at the Unit of Massive Sequencing and Bioinformatic at the Biotechnology Institute, UNAM (UUSMB, IBT), following commands via Security Shell (SSH). The FastQC v. 0.11.8 program and TrimGalore software were used to analyse the sequencing quality. The results of the massive RNA sequencing were deposited in the Bioproject Database (PRJNA1345766; https://www.ncbi.nlm.nih.gov/bioproject/?term=PRJNA1345766).

## Preparation of cell-free extract for enzyme measurements

Mycelia were recovered at different fermentation times by centrifugation and washed twice with extraction buffer (100 mM Tris-HCl (pH 7.3), 5 mM EDTA, 1.2 mM PMSF, and 0.4 mM DTT). The pellet was resuspended in a minimal volume of the same buffer and sonicated at 4 °C for 1 min at intervals of 10 s, with 10 s of rest while it was chilled, using a Soniprep 150MSE sonicator (MSE, London, UK). Cell debris was removed by centrifugation at 22,000×g for 15 min. The supernatant was used for the enzyme activity assays. The protein concentration was determined by the Bradford method (Bradford 1976).

## ATP-dependent glucose kinase assay

ATP-dependent glucose kinase activity was determined spectrophotometrically by monitoring the NADPH concentration at 340 nm according to Mahr et al. (2000), with minor modifications, in a 1-mL cuvette at 25 °C in a standard assay mixture containing 100 mM Tris–HCl (pH 7.5), 5.0 mM MgCl_2_, 0.5 mM NADP^+^, 7.5 mM glucose, 1 mM ATP, and 1 U of glucose 6-phosphate dehydrogenase from *Leuconostoc mesenteroides* (Roche).

## Polyphosphate-dependent glucose kinase assay

Polyphosphate-dependent glucose kinase activity was determined spectrophotometrically by monitoring the NADPH concentration at 340 nm according to the methods of Koide et al. (2013), with minor modifications, in a 1-mL cuvette at 25 °C in a standard assay mixture containing 50 mM Tris-HCl (pH 9.0) with 10% glycerol, 2 mM sodium hexametaphosphate (Sigma‒Aldrich), 1 mM ATP, 5 mM MgCl_2_, 0.5 mM NADP^+^, and 1 U of glucose 6-phosphate dehydrogenase from *Leuconostoc mesenteroides* (Roche).

## Phosphoglucoisomerase assay

The reduction of the β-NADP^+^ intermediate via glucose-6-phosphate dehydrogenase was detected at room temperature by an increase in the absorbance at 340 nm. The assay mixture contained 0.1 mM Tris–HCl buffer (pH 7.6), 2 mM EDTA, 0.5 mM β-NADP^+^, 1 mM fructose-6-phosphate, and 1 U of glucose-6-phosphate dehydrogenase in a final reaction volume of 1 ml (Mathur et al. 2005).

## Phosphofructokinase assay

Fructose-6-phosphate kinase activity was measured spectrophotometrically by coupling fructose-1,6-biphosphate formation to the oxidation of NADH with the use of aldolase, triose phosphate isomerase, and α-glycerophosphate dehydrogenase. The assay solution consisted of 0.1 M Tris-HCl buffer (pH 8.2), 0.2 mM NADH, 1 mM sodium ATP, 10 mM MgCl_2_, 2 mM NH_4_Cl, a mixture of 40 pg of aldolase, 3 pg of triose phosphate isomerase, 30 pg of glycerophosphate dehydrogenase, and 1 mM fructose 6-phosphate in a volume of 1 ml (Babul 1978).

## Aldolase assay

The aldolase assay was carried out by the spectrophotometric method according to Götz et al. (1980). The reaction mixture contained 0.1 M Tris-HCl buffer (pH 7.5), 0.3 mM NADH, 10 mg of glyceraldehyde-3-phosphate dehydrogenase, triosephosphate isomerase and 2.5 mM fructose-1,6 biphosphate in total volume of 1.0 ml.

## Glyceraldehyde 3-phosphate dehydrogenase (GAPDH) assay

GAPDH activity was monitored at 340 nm according to the methods of Maurer et al. (1983), with minor modifications. The 1-ml reaction mixture contained 2 mM NAD^+^ and 50 mM Na_2_HPO_4_ or 4 mM Na_2_HAsO_4_ in 0.1 M Tris-HCl buffer (pH 8.5). Glyceraldehyde 3-phosphate was added to a final concentration of 0.5 mM to start the reaction.

## Phosphoglycerate mutase assay

Phosphoglycerate mutase activity was determined in cell-free extracts. A coupled spectrophotometric assay was carried out in which the product of the first reaction catalysed by phosphoglycerate mutase, namely, 1,3-bisphosphoglycerate, is reduced by the second enzyme, glycerol-3-phosphate dehydrogenase, which uses NADH. Assays were conducted in 0.1 M Tris-HCl buffer (pH 7.4) containing 1 mM EDTA, 2 mM MgSO_4_, 1 mM ATP, 10 mM 3-phosphoglyceric acid, 0.2 mM NADH, and 10 U glycerol-3-phosphate dehydrogenase (Reddy and Wendish 2014).

## Enolase assay

The activity of enolase was determined by direct monitoring of the increase in phosphoenolpyruvate absorbance at 240 nm using a spectrophotometer (Ultrospec 3000; Pharmacia Biotech). The standard assay contained 100 mM Tris (pH 8.0), 0.1 M KCl, 0.5 mM 2-phosphoglycerate and 1 mM MgSO_4_ (Krucinska et al. 2019).

## Pyruvate kinase assay

The initial velocities of the enzyme reaction were assayed spectrophotometrically by coupling the formation of pyruvate from phosphoenolpyruvate and ADP to the reaction of lactate dehydrogenase following the decrease in the absorbance of NADH at 340 nm. All reactions were performed at 25 °C. The reaction mixture contained 100 mM Tris-HCl (pH 7.5), 100 mM KCl, 20 mM MgCl_2_, 0.1 mM NADH, and 6 units of lactate dehydrogenase (Noy et al. 2016).

## Pyruvate dehydrogenase assay

A reaction mixture (1 mL) containing 100 mM Tris-HCl (pH 7.6), 0.2 mM coenzyme A, 0.3 mM thiamine pyrophosphate, 3 mM L-cysteine, 5 mM MgCl_2_, 2 mM NAD^+^ and 3 mM pyruvate was used, and the absorbance at 340 nm was recorded (Komine-Abe et al. 2021).

## Statistical analysis

The EdgeR normalized reading count and specific enzyme activities were determined from three independent biological replicates. Enzyme determinations were also performed in duplicate. The data are shown as the mean ± standard deviation and a two-way ANOVA was performed, considering gene (with 32 levels) and time (with three levels 24 h, 48 h and 72 h) as factors.

## Results and discussion

**Fig. 1:**
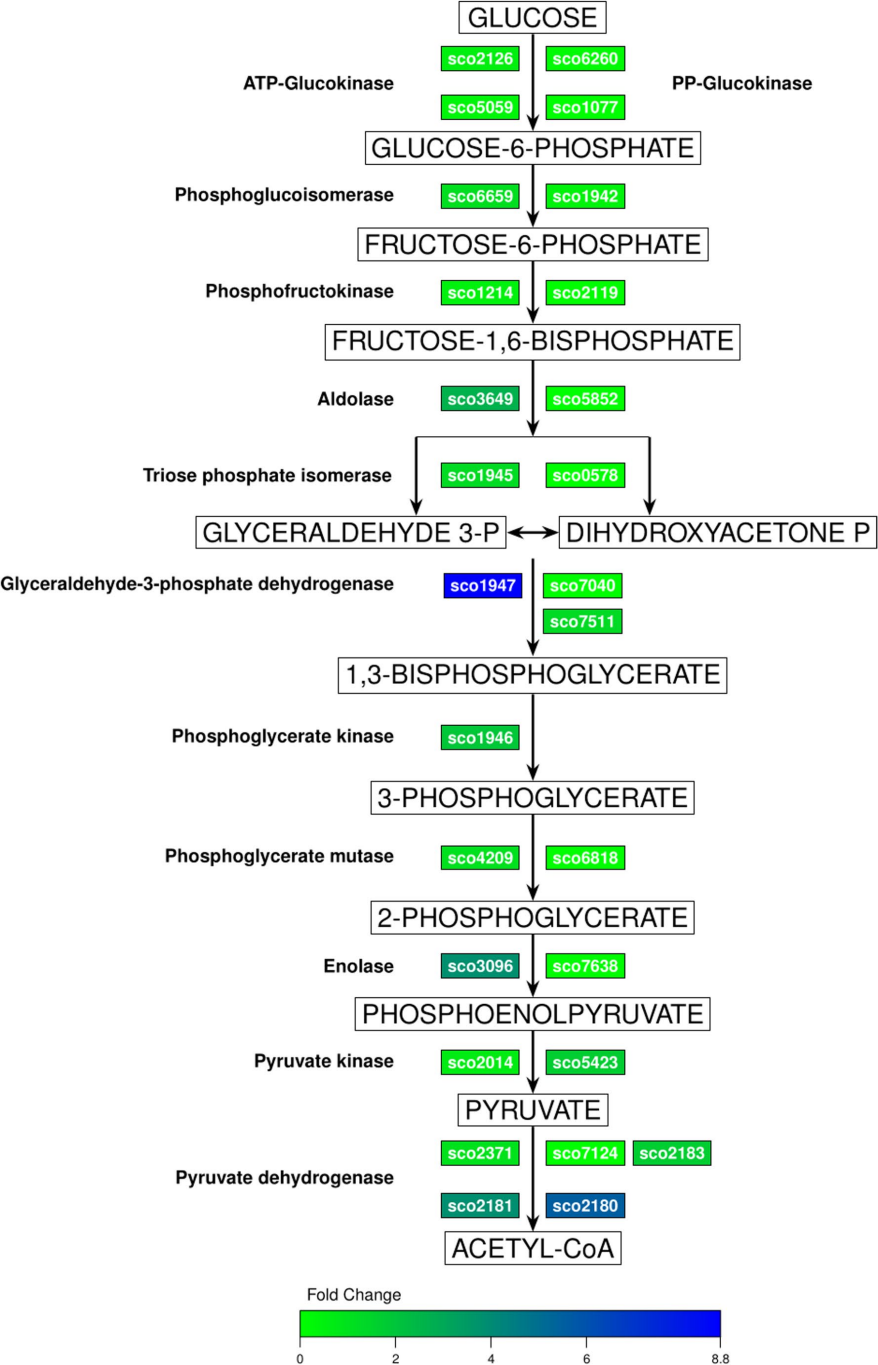
The Glycolytic pathway in *S. coelicolor* M145 with genes encoding enzymes. The intensity of the color indicates the level of expression relative to *hrdB*

The ten enzymes involved in the glycolytic pathway are encoded by 24 genes, of which 7 are located on the left arm of the chromosome and the rest are in the core of the chromosome. As shown in Fig. 1, there is only one gene for phosphoglycerate kinase and two for phosphoglucoisomerase, aldolase, triosephosphate isomerase, enolase and pyruvate kinase. The activities of phosphoglucokinase and glyceraldehyde 3-phosphate dehydrogenase are encoded by three homologous genes, and 4 genes are associated only with phosphoglycerate mutase according to the KEGG database (https://www.genome.jp/kegg/). Thus, *Streptomyces coelicolor*, like many other microorganisms, exhibits gene multiplicity (Schniette et al. 2018); however, whether all or only some of the genes that code for the glycolytic pathway are expressed has not been determined. Massive RNA sequencing allows for the detection of mRNAs at a given time in microorganisms, as well as their relative expression with respect to a reference gene such as the housekeeping sigma factor *hrdB* (Šmídová et al. 2019). It is also not known whether the expression of some of these genes depends on the growth phase of the microorganism or whether the kinetic properties of the enzymes are different (Borodina et al. 2008).

### Glucose transport

*Streptomyces coelicolor* grew on glucose as the sole carbon source, showing exponential growth during the first 48 h, after which it entered the stationary phase; glucose was consumed rapidly during the first 24 h, after which the rate of utilization decreased, as shown in Supplementary Fig. 1. The first step in glucose catabolism is its transport into the mycelium. The presence of two genes encoding proteins belonging to the major facilitator superfamily, *sco5578* and *sco7153* (*glcP1*, *glcP2*; Bertram et al. 2004), has been reported. The expression of *sco5578* relative to that of *hrdB* was just more than twice that of *sco7123*, as reported previously by van Wezel et al. (2005). Both genes have a different dyad repeat which could explain the differences in the transcription of these genes. Notably, these two genes differ by only one nucleotide, resulting in the production of an identical protein, which suggests the occurrence of gene duplication (Bertram et al. 2004).

### ATP-glucose kinase [EC:2.7.1.2] and polyphosphate glucose kinase [EC:2.7.1.63]

**Fig. 2:**
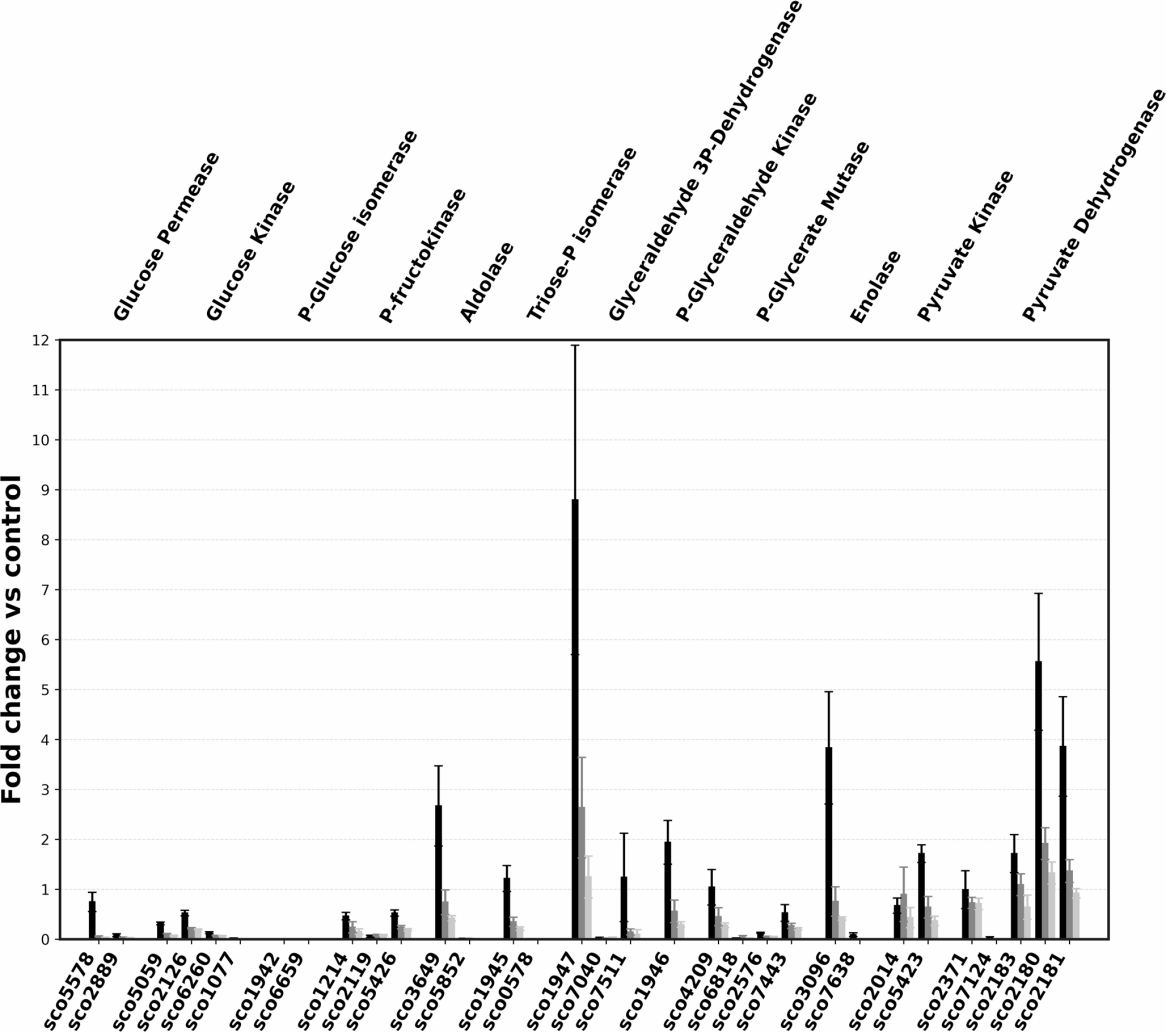
Relative expression to *hrdB* of genes involved in the glycolytic pathway in *S. coelicolor* M145 grown at different times in MM with 1% glucose as sole carbon source (■ 24 h; ■ 48 h; ■ 72 h)

**Fig. 3:**
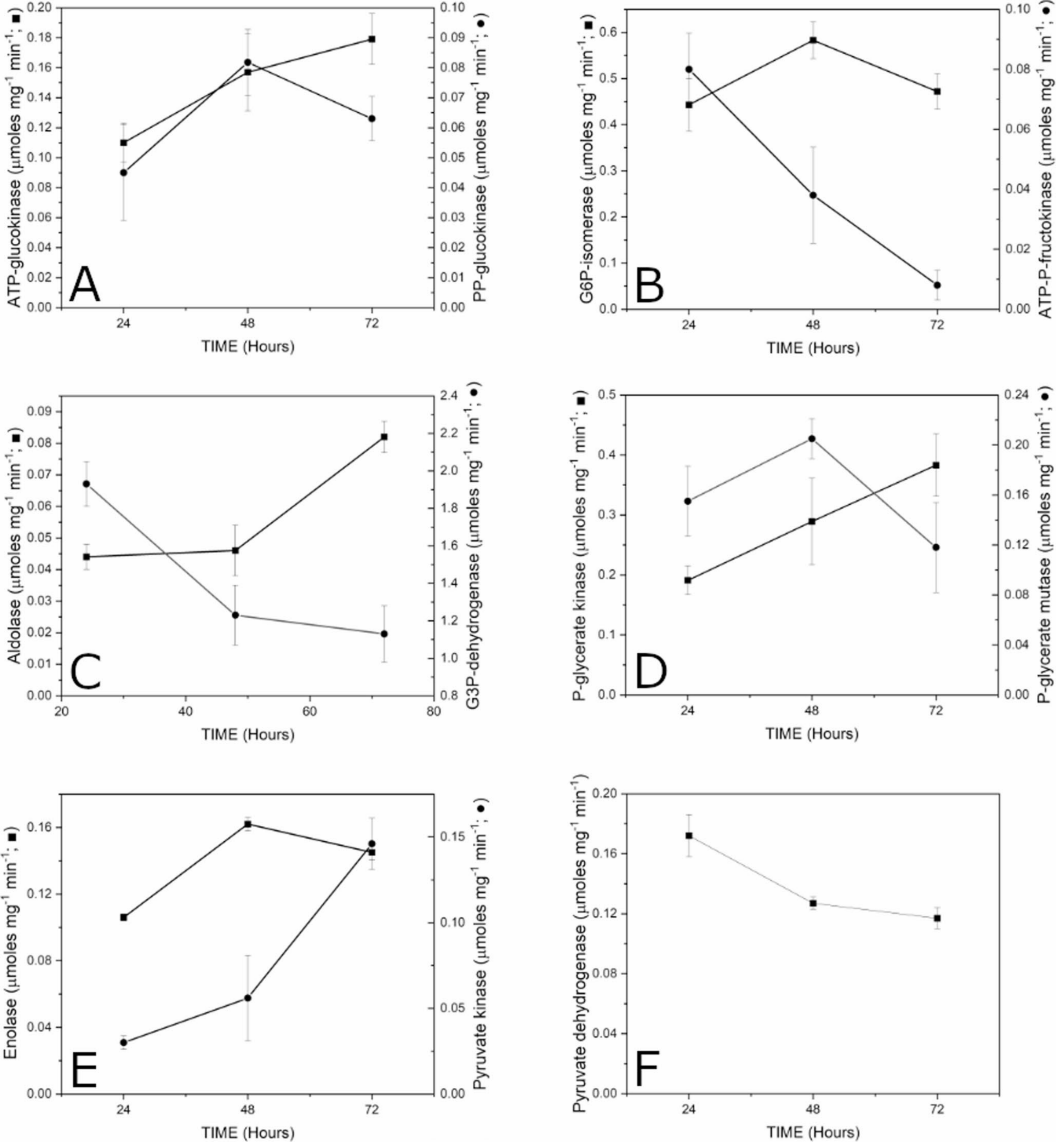
Specific activities of glycolytic pathway enzymes of *S. coelicolor* M145 grown at different times in MM with 1% glucose as a sole carbon source. The enzyme activities were performed as described in Materials and Methods

The first step of glycolysis is the phosphorylation of glucose by glucokinase, and the presence of ATP-dependent and polyphosphate-dependent Glk has been reported (Kawai et al. 2005). Five genes encoding probable glucokinases were detected on the *S. coelicolor* chromosome, *sco2126*, *sco1077*, *sco6260*, *sco6110* and *sco0063* (KEGG). However, from the multiple alignment of the proteins encoded by these genes, it probably can be ruled out that the *sco0063* and *sco6110* genes generate active enzymes since they do not have ATP binding sites (Supplementary Fig. 1). As shown in Fig. 2, the expression of the *sco2126* gene was greatest at 24, 48, and 72 h, followed by that of *sco5059* and finally that of *sco6260*. With respect to these three genes, the highest relative expression occurred at 24 h, after which it decreased, although the expression of *sco2126* decreased by only 34% compared with that at 24 h. The expression of *sco6110* and *sco0063*, also annotated as probable glucokinases, was very low. On the other hand, ATP-Glk activity tended to increase to 72 h, and the activity of PP-Glk peaked at 48 h and decreased slightly at 72 h (Fig. 3; Panel A). This enzyme activity has been attributed to the ATP-Glk encoded by *sco2126*, although it cannot be ruled out that the activities of the enzymes encoded by *sco1077* and *sco6260* were also included (Imriskova et al. 2005), and PP-Glk was associated with *sco5059* (Koide et al. 2013). The ATP-Glk-specific activity obtained was much lower than that reported by Mahr et al. (2000); however, the time points at which cell-free extracts were obtained for quantification of the activity were very different between our study and the Mahr et al. (2000) study, which could explain these variations. The identity between all the glucokinase proteins was between 21 and 38% (Supplementary Fig. 2).

### Glucose 6-phosphateisomerase [EC:5.3.1.9]

The genes *sco1942* and *sco6659* are associated with phosphoglucoisomerase activity, which catalyses the reversible isomerization of D-glucopyranose-6-phosphate and D-fructofuranose-6-phosphate and plays a central role in glycolysis and gluconeogenesis (Mathur and Garg 2007). The relative expression of *sco6659* was 2.5-fold greater than that of *sco1942*, and the expression of both genes tended to decrease over time to approximately 10% (Fig. 2), while the activity of phosphoglucoisomerase increased from 24 to 48 h but then decreased thereafter (Fig. 3; Panel B). The identity of the proteins encoded by these two genes was 90% (Supplementary Fig. 3). To the best of our knowledge, this enzyme has not been studied in *Streptomyces*, much less its expression in a minimal medium with glucose as a carbon source. However, it is not very common to find two genes that encode isoenzymes with phosphoglucoisomerase activity in *Streptomyces* (*Streptomyces hygroscopicus* and *Streptomyces avermitilis*) since the majority have a single gene (Takahashi et al. 2022).

### ATP-dependent 6-Phosphofructokinase [EC:5.3.1.9]

Phosphofructokinase catalyses the phosphorylation of fructose-6-phosphate to fructose-1,6-bisphosphate, a key regulatory step in the glycolytic pathway. Three genes (*sco1214*, *sco2119*, and *sco5426*) on the *S. coelicolor* chromosome are associated with this enzymatic activity (Borodina et al. 2008). The most highly expressed gene relative to *hrdB* was *sco5426*, followed by *sco1214* and finally *sco2119*, whose expression tended to increase, reaching a maximum at 72 h (Fig. 2), whereas the expression of *sco5426* and *sco1214* decreased over time. Specific activity peaked at 48 h and subsequently decreased (Fig. 3; Panel C). The phosphofructokinases SCO1214 and SCO5426 share 72% identity, whereas SCO2119 is less similar to the other two (Supplementary Fig. 4). It has been reported that the deletion of *sco5426* increases the flow of carbon through the pentose phosphate pathway, increasing the production of the pigmented antibiotics actinorhodin and undecylprodigiosin, which suggests that the activity of phosphofructokinase is due mainly to the transcription of this gene (Borodina et al. 2008). Hesketh et al. (2002) reported the presence of the Pfk2 and Pfk3 proteins in a proteomic study, which is in agreement with our results concerning the greater relative expression of the mRNAs corresponding to *sco542*6 and *sco1214* in *S. coelicolor* grown in the presence of glucose.

### Fructose-biphosphatealdolase [EC:4.1.2.13]

The fructose 1,6-bisphosphate generated by the above reaction is converted into glyceraldehyde 3-phosphate and dihydroxyacetone phosphate by the action of aldolase. Two genes are annotated in the genome for this enzymatic activity, *sco3649* and *sco5852*, of which the former is predominantly expressed, whereas the latter is expressed only at basal levels (Fig. 2). The specific activity of aldolase was similar at 24 and 48 h and increased by 50% at 72 h (Fig. 3; Panel D). The protein products of these two genes share 25% identity (Supplementary Fig. 5). Aldolase is indispensable for glycolysis; however, very little is known about the expression or activity of this enzyme in actinomycetes.

### Triosephosphate isomerase [EC:5.3.1.1]

The interconversion between glyceraldehyde 3-phosphate and dihydroxyacetone phosphate is carried out by a triosephosphate isomerase, which is likely encoded by the genes *sco1945* and *sco0578*, of which only the former is expressed, as *sco0578* showed a relative expression close to zero (Fig. 2). The specific activity of this enzyme remained constant between 24 and 48 h but increased to 72 h. The identity between the two proteins encoded by these genes was 32% (Supplementary Fig. 6).

### Glyceraldehyde 3-phosphate dehydrogenase [EC:1.2.1.12]

One of the important steps of the EMP pathway is the activity of NAD-dependent glyceraldehyde-3-phosphate (GAP) dehydrogenase, which affects both glycolysis and gluconeogenesis because of the reversibility of the reaction it catalyses. *sco1947*, which encodes a glyceraldehyde 3-phosphate dehydrogenase in *S. coelicolor*, had the highest relative expression of all the genes associated with the glycolytic pathway. Its relative expression subsequently decreased (Fig. 2). Of the other two genes annotated for this activity, *sco7040* and *sco7511*, only the latter showed a relative expression that was 8.6-fold lower than that of *sco1947* and decreased over time. In antibiotic-producing *Streptomyces*, two to four genes encoding glyceraldehyde 3-phosphate dehydrogenase can be found (Takahashi et al. 2022). On the other hand, in the actinomycete *Corynebacterium glutamicum*, two genes, *gapA* and *gapB*, have been reported to generate two proteins of different molecular weights, the first of which displayed activity in glycolysis and the second in gluconeogenesis (Omumasaba et al. 2004). *S. coelicolor* has two small enzymes and one large enzyme; the *sco7040* gene, which encodes the 52.4 kDa protein, is poorly expressed, but whether *sco7040* can be expressed under gluconeogenic conditions is not yet known. The specific activity of this enzyme in *S. coelicolor* was also much greater than that of the other glycolytic enzymes, decreasing to 1.2 µmol ml^− 1^ min^− 1^ and remaining virtually constant from 48 to 72 h; this activity level is 10 times greater than that in *C. glutamicum* grown in minimal medium with glucose as a carbon source (Fig. 3; Panel E). The highest identity was found between the SCO1947 and SCO7511 proteins, at 57% (Supplementary Fig. 7).

### Phosphoglyceratekinase [EC:2.7.2.3]

Phosphoglycerate kinase is involved in the first ATP-generating step of the glycolytic pathway and is the only enzyme in the pathway for which there is only one annotated gene in the *S. coelicolor* genome (*sco1946*). In other *Streptomyces* species, it is also a unique gene, perhaps because of the importance of phosphorylation at the substrate level. The relative mRNA expression of this gene was intermediate and decreased over time (Fig. 2). The specific activity increased linearly from 24 to 72 h and this enzyme was the only one that exhibited this behaviour (Fig. 3, Panel F). Surprisingly, despite the importance of this enzymatic step in the EPM pathway, almost no information is available concerning the expression of this gene or its enzymatic activity in actinomycetes.

### 2,3-biphosphoglycerate-dependent phosphoglycerate mutase [EC:5.4.2.11]

Phosphoglycerate mutase catalyses the isomerization of phosphoglycerate substrates, a process essential for the metabolism of glucose and/or 2,3-phosphoglycerate. It is encoded by two genes, *sco4209* and *sco6818*, according to KEGG. The relative expression of *sco4209* started high and decreased over time, whereas that of *sco6818* was very low (Fig. 2). The time-dependent activity profile of this enzyme peaked at 48 h and subsequently decreased (Fig. 3, Panel G). The proteins derived from these two genes exhibit very low identity and similarity, then probably the protein encoded by *sco6818* was not actually a phosphoglycerate mutase (Supplementary Fig. 8). Purification of phosphoglycerate mutase from *S. coelicolor* has been reported, and according to the amino acid sequence and molecular weight of the protein, it corresponds to the product of *sco4209*, but neither the expression of this gene nor its regulation has been reported (White et al. 1992).

### 2-Phosphoglycerate hydrolase [EC:4.2.1.11]

2-Phosphoglycerate hydrolase, or enolase, is a metalloenzyme that catalyses the reversible elimination of water from 2-phosphoglycerate to form phosphoenolpyruvate during glycolysis (Kumar et al. 2023). Two genes, *sco3096* and *sco7638*, are associated with this enzymatic activity in *S. coelicolor*. The relative expression of *sco3096* started high and tended to decrease up to 72 h, while that of *sco7638* was very low (Fig. 2). The specific activity of enolase in the cell-free extracts of this microorganism significantly increased to 48 h but decreased thereafter (Fig. 3, Panel H). The identity between the two proteins encoded by these genes was 57% (Supplementary Fig. 10). There is no information about the expression of the genes that encode enolase or its physicochemical properties in *Streptomyces*, but genome sequencing has shown that they may have one or two genes that encode enolase.

### Pyruvate kinase [EC:2.7.1.40]

Pyruvate kinase catalyses the last step in glycolysis encoded by two genes, *sco2014* and *sco5423*, whose relative expression was 2.5 times greater than that of *sco2014* at 24 h, with both decreasing up to 72 h (Fig. 2). In contrast, the activity of this enzyme tended to increase from 24 to 72 h (Fig. 3, Panel I). The identity of the two proteins was almost 69%, and the similarity was 83% (Supplementary Fig. 10).

### Pyruvate dehydrogenase [EC:1.2.4.1] [EC:2.3.1.12] [EC:1.8.1.4]

Pyruvate dehydrogenase links glycolysis to the tricarboxylic acid cycle. Pyruvate dehydrogenase is a multienzyme complex that consists of three enzymes: E1 (which uses thiamine pyrophosphate as its cofactor), E2 (which uses lipoamide as its cofactor), and E3 (which uses flavin adenine dinucleotide as its cofactor) (Škerlová et al. 2021). The E1 subunit is encoded by the genes *sco2371*, *sco2183*, and *sco7124*, of which the relative expression of the first two was similar and tended to decrease over time, while *sco7124* was essentially not expressed. The relative expression of *sco2181*, which encodes the E2 subunit, was intermediate and decreased over time, whereas that of *sco2183* was the highest and decreased over time. Notably, *sco2180*, *sco2181*, and *sco2183* are physically located close together in the *S. coelicolor* genome. *sco2182* encodes a putative gntR family transcriptional regulator. The relative expression differed among the four genes, suggesting that they are transcribed independently from their own promoters. Notably, the E2 and E3 subunits are shared with the α-ketoglutarate dehydrogenase multienzyme complex, which could explain why the corresponding genes were more highly expressed than the other genes were. Pyruvate dehydrogenase and α-ketoglutarate dehydrogenase from *Corynebacterium glutamicum* also share the same CgE2 (AceF) and CgE3 (Lpd) (Komine-Abe et al. 2021). The pyruvate dehydrogenase activity profile tended to decrease as a function of growth time in *S. coelicolor*, with activity levels 10-fold higher than those of α-ketoglutarate dehydrogenase under the same growth conditions (data not shown).

The results of this study showed that the presence of paralogous genes for the same enzymatic activity does not imply that their transcription is equal. In most cases, one of them will have higher mRNA levels or only one will be transcribed. Gene position on the chromosome also plays no role, as *sco1947*, located on one of the arms, was the most highly expressed of all. Notably, although some groups of genes are physically together (*sco1945*, *sco1946* and *sco1947*), each has its own promoter since their mRNA levels differed (Fig. 2). The transcription levels of most genes encoding enzymes in the glycolytic pathway tended to decrease over time. According to the growth curve, at 24 h, *S. coelicolor* was in the exponential phase of growth, whereas at 48 h, it was entering the stationary phase, which explains why mRNA levels decreased; at 72 h, it was already in the stationary phase.

The results of the two-way ANOVA statistical analysis showed that both gene and time had highly significant effects on the variable (*p* < 0.001 in both cases). In addition, a highly significant gene × time interaction was observed (*p* < 0.001), so the temporal behavior was not uniform for all genes involved in the glycolytic pathway in *S. coelicolor* (Supplementary Table 1). On the other hand, compared with the other genes, ATP-glucokinase, despite its importance in glucose phosphorylation, was expressed at low levels. Furthermore, Glk, encoded by *sco2126*, was the most highly expressed paralogue in this study and is involved in glucose catabolic repression (Ruiz-Villafán et al. 2021).

On the other hand, the activity of the enzymes involved in this pathway showed three different trends depending on the growth period. PP-Glk, glucose 6-phosphate isomerase, phosphoglycerate mutase, and enolase peaked at 48 h, corresponding to the end of growth, whereas ATP-Glk, aldolase, phosphoglycerate kinase, and pyruvate kinase tended to increase until the stationary phase. The specific activities of ATP-phosphofructokinase, glyceraldehyde 3-phosphate dehydrogenase, and pyruvate dehydrogenase decreased steadily from 24 to 72 h of growth.

Expression profiles generally decreased, but specific enzyme activity profiles peaked at 48 h, or, like that of aldolase, increased from 48 to 72 h. These discrepancies are difficult to explain because expression is influenced by promoter strength, mRNA stability, and transcription regulation, whereas enzyme activity depends on protein stability, including its half-life and post-translational modifications as it has been reported by Yang et al. (2021).

## Conclusion

The relative mRNA levels of genes involved in the enzymatic steps of the glycolytic pathway in *Streptomyces coelicolor* M145 differed, with the highest being *sco1947*, which encodes glyceraldehyde 3-phosphate dehydrogenase.

One of the genes with the lowest relative expression levels and low specific activity was *sco2126*, which encodes an ATP-dependent glucose kinase. This step is likely limiting carbon flow in this microorganism.

Gene multiplicity does not imply that all paralogous genes are transcribed at the same level. In most cases, one gene is always preferentially transcribed.

The large differences in gene expression and enzyme activity involved in the glycolytic pathway of *Streptomyces coelicolor*, as well as the identification of the limiting steps, may allow the design of strategies aimed at improving carbon flow in this microorganism.

## Supplementary Information

Below is the link to the electronic supplementary material.

Supplementary Material 1(PDF 678 KB)

Supplementary Material 2(PDF 35.4 KB)

## Data Availability

Massive RNA sequencing were deposited in the Bioproject Database (PRJNA1345766; https://www.ncbi.nlm.nih.gov/bioproject/?term=PRJNA1345766).
