# Supplementary material for: Transcriptional analysis of genes associated with glycolysis in Streptomyces coelicolor M145
Source: Int Microbiol. 2025 Nov 4;28(8):3053–63. doi: 10.1007/s10123-025-00744-6 (PMC12727718; doi:10.1007/s10123-025-00744-6)
Supplement: Supplementary file 1 — Supplementary Material 1(PDF 678 KB) [file 10123_2025_744_MOESM1_ESM.pdf]

Supplementary figures

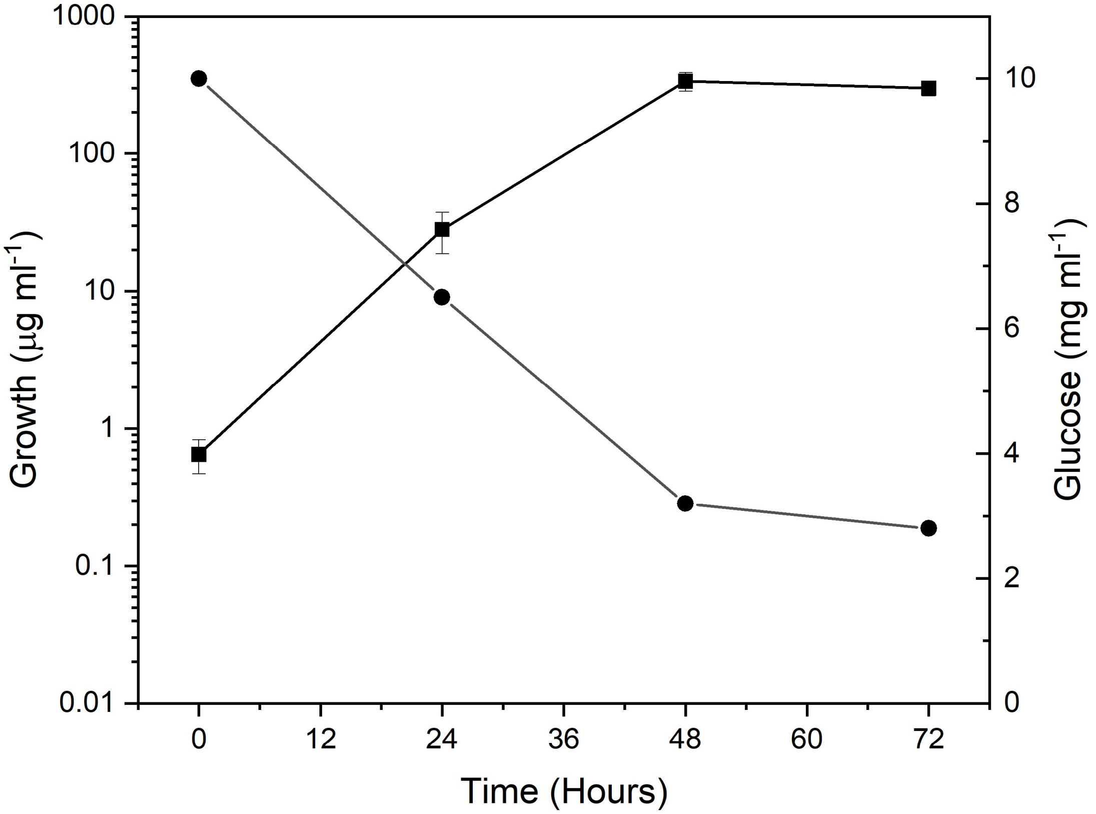

Supplementary Fig 1. Biomass (■) and residual glucose (●) of *Streptomyces coelicolor* M145 grown in MM with 1% glucose. Samples were taken at the indicated times, and growth was quantified as described in the "Materials and methods" section. Bars represent the standard deviation, and all measurements were performed at least in triplicate.

Figure made with the Origin program

```
SCO5059            -----------------------------------MQIFGVDIGGSGIKGAPVDLDRGDL 25
SCO0063            ------------------MIT-------ATTRHASRPVPVLDIGGTHVTAALVDPTASRP 35
SCO4285            -----------------------------------------------MKAALAGPGGELL 13
SCO6110            -----------------------------MSGPGAPVFAGVDIGGTTTQVVLCDEELKVL 31
SCO1077            --------------------------------MHTDLVAALDIGGTKIAGALVDGHGRIQ 28
SCO6260            MSTYRDFTAPIGSRRAPVLRTVGTRERRSHLTAPRVPTVGIDIGGTKVMAGVVDADGNIL 60
SCO2126            ----------------------------------MGLTIGVDIGGTKIAAGVVDEEGNIL 26
3VGM_1|SgGlk|      ----------------------------------MGLTIGVDIGGTKIAAGVVDEEGRIL 26
                                                                        .

SCO5059            AQER-CKVLTPHPATPD----GVADGVKQVVEH-----FGWTGRVGLTFPGVVTGGATVR 75
SCO0063            VPSPVIRRPLRAHADAD----AVLDAIARAALA--LP-AGHSPRWGVAMPGPFDYATGVG 88
SCO4285            HQA---RRATGRERGPEAVVAGILDFAAELRAYGADRFGEPARAAGVAVPGIVDAERGVA 70
SCO6110            DRA---DTATPARSGGRAMIAATLDALAV--VR--ARTPGRLAAVGVGAAGVVDAASGHI 84
SCO1077            ARA---QRATPAREDGDTVMRAVEDVLAELTV---SPLWGRASAVGIGSAGPVDASAGTV 82
SCO6260            EKL---RTETPDKSKSPK---VVEDTIVELVLD--LSDRHDVHAVGIGAAGWVDADRNRV 112
SCO2126            STH---KVPTPTT---PE---AIVDAIASAVEG--ARVGHEIVAVGIGAAGYVNRQRSTV 75
3VGM_1|SgGlk|      STF---KVATPPT---AE---GIVDAICAAVAG--ASEGHDVEAVGIGAAGYVDDKRATV 75
                                           *                    *:   * .

SCO5059            TAAN--VDKGWV----DTDARALFAERLGGLDVTVVNDADAAGVAEMHFGAGRGRKGTVV 129
SCO0063            RFADVGKFESLNGVDVGAGLRERLADRC--ERLRFLNDADAFAVGEYGSGAAAGHD-HVV 145
SCO4285            VYAA--NL-GWR----DVPLRTLLGERLGSIPVALGHDVRTGGLAEGRIGAGRGAD-RFL 122
SCO6110            LVASDSFT-DWA----GFPVTAALRTA-LGVPAFLDNDVNAFLRGEIACGAAADEE-DVL 137
SCO1077            SPVN--VP-GWR----DYPLVRRVQAAAGGLPVELIGDGVAITAAEHWQGAARGHD-NAL 134
SCO6260            LFAP--HL-SWR----NEPLRDRIAGR-LAVPVLVDNDANTAAWAEWRFGAGRGED-HLV 163
SCO2126            YFAP--NI-DWR----QEPLKEKVEAR-VGLPVVVENDANAAAWGEYKFGGGKGHR-NVI 126
3VGM_1|SgGlk|      LFAP--NI-DWR----HEPLKDKVEQR-VGLPVVVENDANAAAWGEYRFGAGQGHD-DVI 126
                     .      .             .          .  *  :   .*   *.. .     :

SCO5059            LLTFGTGIGSAVFTDGLLVPN------TELGHLELD--GHDA---------EKRASS--- 169
SCO0063            CLTLGTGVGSSFLSGGRPVHSGPL----------VPPGGHAYRLTVHGRPLEDTVSRRGI 195
SCO4285            FVPLGTGIAGAIGIDGRVEA-GAHGFAGEIGHVVVRPGGIACPCGQRG-CLERFASASAV 180
SCO6110            GMTLGTGVGGALWTGGELRT-GAHGAAGEIGHIPG-FGDLPCTCGGRG-HLETLASARSL 194
SCO1077            CMVVSTGVGGGLVLGGRLHP-GPTGNAGHIGHISVDLDGDPCPCGARG-CVERIASGPNI 192
SCO6260            MITLGTGIGGAILEDGQVKR-GKYGVAGEFGHMQVVPGGHRCPCGNRG-CWEQYSSGNAL 221
SCO2126            CITLGTGLGGGIIIGNKLRR-GHFGVAAEFGHIRMVPDGLLCGCGSQG-CWEQYASGRAL 184
3VGM_1|SgGlk|      CITLGTGLGGGIIIGNKLRR-GRFGVAAEFGHIRVVPDGLLCGCGSQG-CWEQYASGRAL 184
                    : ..**:....  ..                      .            *   *

SCO5059            ------------------------------------KVKDD---HDMSWEHWAHRVQKYL 190
SCO0063            RAHYARLT------------SATGDQHLPDVRDIAARARKGDAAAQEAFRYAFDALGQAL 243
SCO4285            SQAWAQAC----------------GDPGADAADCAKAVESGDARALAVWQDAVDALADGL 224
SCO6110            RARYGER-----------------TGRELTAREVAEAARDGDADAHGVFAAAGAGIARAI 237
SCO1077            ARRALEAG------------WLPGPDGDTSAAAVAASARLGDPVAVASFERAAQALAAGI 240
SCO6260            VREARELAAADSPVAYGIIEHVKGSIGDITGPMITELAREGDAMCVELLQDIGQWLGVGI 281
SCO2126            VRYAKQRANATPERAEVLLALGDGTPDGIEGKHISVAARQGCPVAVDSYRELARWAGAGL 244
3VGM_1|SgGlk|      VRYAKQRANATPENAAVLLGLGDGSVDGIEGKHISEAARQGDPVAVDSFRELARWAGAGL 244
                                                        .. .                  :

SCO5059            AHVEMLFSPELFIIGGGVSRKSQKFLPHIKDV------------RAEIVPAQLQNNAGIV 238
SCO0063            APWIDRFEATAVVIGGSMARSWDLIHPALTTGRAP---A--DGPDVPVLPARQPEEAPLL 298
SCO4285            VTALTLLDPRVLIIGGGLAEAGETLFTPLRDAVRRRVTF---QKLPEIVPAALGDTAGCL 281
SCO6110            VMTAGLVDIGTVVIGGGVSGAWTLLEPLIREPLAAEPPISG--HPVKLLRAALGPDAVPL 295
SCO1077            AATATLVEIDIAVVGGGVGKAGDVLFAPLRKALTDYATL-SFVQRLAVVPAQMGTDAGLV 299
SCO6260            ANLAAALDPSCFVIGGGVSAADDLLIGPARDAFKRQLTGRGYRPEARIVRAQLGPEAGMV 341
SCO2126            ADLASLFDPSAFIVGGGLSDEGDLVLDPIRKSYKRWLVGGNWRPVADVIAAQLGNKAGLV 304
3VGM_1|SgGlk|      ADLASLFDPSAFIVGGGVSDEGELVLDPIRKSFRRWLIGGEWRPHAQVLAAQLGGKAGLV 304
```

```
                     .     ..    ::**.:.     .                       :: *      *  :

SCO5059              GAAMHAAG------------------------------------  246
SCO0063              GAAHWAQGTPDAP-------------------------------  311
SCO4285              GAGLMAWDLLDTAAPSAPSAPSAPSAPSSTASTAATAATPPEVTT  326
SCO6110              GAAARARHELTAR--------------------------------  308
SCO1077              GAAAAALTRTADVTAAHV---------------------------  317
SCO6260              GAADLSRLVARRFRRAKRRRVERYERYERYA--EARRESRESL--  382
SCO2126              GAADLAREPDPIM--------------------------------  317
3VGM_1|SgGlk|        GAADLARQGLEHHHHHH----------------------------  321
                     **.  :
```

Supplementary fig 2 Multiple alignment of amino acid sequences of the probable glucokinases from *S. coelicolor* and Glk from *S. griseus* (pdb_00003vgm). Amino acids involved in the ATP-binding motifs (underlined). The alignment was performed using CLUSTAL O (1.2.4) Multiple sequence alignment.

```
sco1942            1 MNADGRTRLHQTPEWAALVKH-REQLGEVRLREMFAADPGRGTGWTLRVG     49
                       .....:|:|.|||.||..| :..|....|||:||.||||...:.:|||
sco6659            1 --MSDTPKLNQRPEWTALADHAKGTLPHPDLRELFAQDPGRAERYVVRVG     48

sco1942           50 DLHVDYSKHLVTDETLALLQELAAATGVSGLRDAMFRGERINITEDRAVL     99
                     ||.:||||||||||||||||||||||||||||||||||||||||||||||
sco6659           49 DLRIDYSKHLVTDETLALLQELAAATGVSGLRDAMFRGERINITEDRAVL     98

sco1942          100 HTALRAPRDAVIEVDGENVVPQVHAVLDKMAGFADRVRTGEWTGHTGRRI    149
                     ||||||||||||||||||||||||||||||||||||||:|||||||||||
sco6659           99 HTALRAPRDAVIEVDGENVVPQVHAVLDKMAGFADRVRSGEWTGHTGRRI    148

sco1942          150 RNVVNIGIGGSDLGPAMAYEALRAFTDRSLTLRFVSNVDGADLHEAVRDL    199
                     ||||||||||||||||||||||||||||||||||||||||||||||||||
sco6659          149 RNVVNIGIGGSDLGPAMAYEALRAFTDRSLTLRFVSNVDGADLHEAVRDL    198

sco1942          200 DPAETLFIIASKTFTTIETITNATSARSWLLAGLGGDEKAVAKHFVALST    249
                     |||||||||||||||||||||||||||||||.||||||||||||||||||
sco6659          199 DPAETLFIIASKTFTTIETITNATSARSWLLDGLGGDEKAVAKHFVALST    248

sco1942          250 NAEKVADFGIDTANMFEFWDWVGGRYSFDSAIGLSLMIAIGPDRFREMLD    299
                     ||||||||||||||||||||||||||||||||||||||||||||||||||
sco6659          249 NAEKVADFGIDTANMFEFWDWVGGRYSFDSAIGLSLMIAIGPDRFREMLD    298

sco1942          300 GFHLVDEHFRTAPAESNVPLLMGLLGVWYGSFLGAQSHAVLPYSHYLSKF    349
                     ||.:||||||.|.|.:|.|||:||||||||.|||||||||||||||||||
sco6659          299 GFRIVDEHFRNAEAPANAPLLLGLLGVWYGDFLGAQSHAVLPYSHYLSKF    348

sco1942          350 TAYLQQLDMESNGKSVDREGKPVQWQTGPVVWGTPGTNGQHAYYQLIHQG    399
                     ||||||||||||||||||||.|||||||||||||||||||||||||||||
sco6659          349 TAYLQQLDMESNGKSVDREGNPVQWQTGPVVWGTPGTNGQHAYYQLIHQG    398

sco1942          400 TKLIPADFIGFARPVDELSEELKSQHDLLMANFFAQTQALAFGKTPDEVR    449
                     |||||||||||||||||||||||:||||||||||||||||||||||||||
sco6659          399 TKLIPADFIGFARPVDELSEELKAQHDLLMANFFAQTQALAFGKTPDEVR    448

sco1942          450 AEGVPEELVPHKTFPGDHPTTTILARELTPSVLGQLVALYEHKVFVQGAI    499
                     ||||||||||||||.|:|||||:||.||||||||||:|||||||||||||
sco6659          449 AEGVPEELVPHKTFRGNHPTTTVLAAELTPSVLGQLIALYEHKVFVQGAI    498

sco1942          500 WNIDSFDQWGVELGKVLAKRVEPALTEGADVPGLDPSTGALVAAYRELRG    549
                     ||||||||||||||||||||||||||||||||||||||.|||||||||:.
sco6659          499 WNIDSFDQWGVELGKVLAKRVEPALTEGADVPGLDPSTAALVAAYRELKE    548

sco1942          550 RR    551
                     ..
sco6659          549 VH    550
```

Supplementary Fig 3 Pairwise Sequence alignment paired between the protein sequences of *S. coelicolor* M145 phosphoglucoisomerases (SCO1942, SCO6659). The alignment was performed with the EMBOSS Needle program.

```
SCO2119      MKVGVLTGGGDCPGLNAVIRAVVRKGVQEYGYDFTGFRDGWRGPLEGDTVPLDIPAVRGI 60
SCO1214      MRIGVLTSGGDCPGLNAVIRSVVHRAVVDHGDEVIGFRDGWKGLLECDYLKLDLDAVGGI 60
SCO5426      MRIGVLTAGGDCPGLNAVIRSVVHRAVDNYGDEVIGFEDGYAGLLDGRYRALDLNAVSGI 60
             *::****.************:**::.* ::* :. **.**: * *:     **: ** **

SCO2119      LPRGGTVLGSSRTNPLKQRDGIRRIKDNLAALGVEALITIGGEDTLGVATRLADEYGVPC 120
SCO1214      LARGGTILGSSRVRPEHLRDGVERARGHVEELGLDAIIPIGGEGTLKAA-RLLSDNGLPI 119
SCO5426      LARGGTILGSSRLERDRLREACENAGDMIQNFGIDALIPIGGEGTLTAA-RMLSDAGLPV 119
             * ****:***** .  : *:. ..  . :  :*::*:* ****.** .* *: .: *:*

SCO2119      VGVPKTIDNDLSATDYTFGFDTAVGIATEAIDRLHTTAESHMRVLVVEVMGRHAGWIALH 180
SCO1214      VGVPKTIDNDIAVTDVTFGFDTAVTVATEALDRLKTTAESHQRVLIVEVMGRHTGWIALH 179
SCO5426      VGVPKTIDNDISSTDRTFGFDTAVGVATEAMDRLKTTAESHQRVMVVEVMGRHAGWIALE 179
             **********:: ** ******** :****:***:****** **::*******:*****.

SCO2119      SGLAGGANVILIPEQRFDVEQVCSWVTSRFRASY-APIVVVAEGAMPRDGDMVLKDESLD 239
SCO1214      SGMAAGAHAVVVPERPFDIDELTAKVGERFSAGKRFAIVVAAEGAKPKAGTMDFDEGGKD 239
SCO5426      SGMAAGAHGICLPERPFDPADLVKMVEERFSRGKKFAVVCVAEGAHPAEGSMDYGKGAID 239
             **:*.**: : :**: **  ::   * .**  .    :* .**** *  * *   . . *

SCO2119      SYGHVRLSGVGEWLAKQIEKRTGNEARTTVLGHVQRGGTPSAFDRWLATRFGLHAVDCVH 299
SCO1214      VYGHERFAGIARQLSIELEERLGKEARPVILGHVQRGGTPTAYDRVLATRFGWHAVEAVH 299
SCO5426      KFGHERFQGIGTALAFELERRLGKEAKPVILGHVQRGGVPTAYDRVLATRFGWHAVEAAH 299
              :** *: *:.  *: ::*.* *:**: .:********.*:*:** ****** ***:..*

SCO2119      DGDFGKMVALRGTDIVRVPIAEATARLKTVDPALYEEVGVFFG    342
SCO1214      RGEFGKMTALRGTDIEMVSLADAVESLKTVPDARYAEAECVL-    341
SCO5426      RGDFGRMTALRGTDVVMVPLAEAVTELKTVPKDRMDEAESVF-    341
              *:**:*.******:  * :*:*.  ****      *.  .:
```

Supplementary Fig 4 Multiple Sequence alignment paired between the protein sequences of *S. coelicolor* M145 phosphofructokinases (SCO1214, SCO2119, SCO5426). The alignment was performed using CLUSTAL O (1.2.4) Multiple sequence alignment.

```
sco3649            1 MP-IATPEVYNEMLDRAKAGKFAYPAINVTSSQTLHAALRGFAEAESDGI     49
                     || :.|.|:.:|   .|.||: |..|.||.:.:...|...|...|.:..|
sco5852            1 MPLVPTRELVSE---AAVAGR-AVAAFNVITLEHAEAIASGAQAAGAPVI     46

sco3649           50 VQISTGGAEFLGGQHNKDMVTGAVALAEFAHIVAEKYDVTVALHTDHCPK     99
                     :|||.....|.||:        ...:|..|..|.:...|.||||.||   
sco5852           47 LQISENAVRFHGGR--------VEPIARAAAEVGKACGVDVALHLDH---     85

sco3649          100 DKLDGYVRPLIAVSEERVKAG-RNPLFQSHMWDGSAETLADNLSIAQELL    148
                                 |::.|:..| .:..|.|.|:|..|:..|:||:..:...
sco5852           86 ------------VTDPRLLHGAADAGFSSAMFDAGAQPYAENLAATRAAA    123

sco3649          149 ARARAARIILEVEITPTGGEEDGVSHEINDSLYTTVDDAVRTVEALGLGE    198
                     ..|..|.:.||.|:...||:.|..:......:.|...:|.|.|...|:. 
sco5852          124 QWAHGAGLWLEAELGYVGGKPDAPASAHAAGVRTDPQEAARYVADTGVD-    172

sco3649          199 KGRYLLAASFGNVHGVYKPGNVVLRPELLKELNEGIASKYGQPAGSKPFD    248
                         .||.:.|:.|.:.: .:..|...|::.|.|.:           |..
sco5852          173 ----ALAVAVGSSHAMTE-RSASLDHALIERLREAV-----------PVP    206

sco3649          249 FVFHGGSGSTAEEIATALENGVVKMNIDTDTQYAFTRPVVDHMFRNYDGV    298
                     .|.||.||...:|:..|:..|::|:|:.|....|||..|.:.:....|  
sco5852          207 LVLHGSSGVGDDELRRAVRAGILKVNVGTALNIAFTGAVRETLAARPD--    254

sco3649          299 LKVDGEVGNKKTYDPRTWGKLAEAGMA----ARVVEACGHLRSAGQKIK    343
                                ..|||.:.......||    |.:....|       
sco5852          255 -----------LTDPRPYVARGREAMAETVRALLAVVSG----------    282
```

Supplementary Fig 5 Pairwise Sequence alignment paired between the protein sequences of *S. coelicolor* M145 aldolases (SCO3649, SCO5852). The alignment was performed with the EMBOSS Needle program.

```
sco1945            1 --MTTRTPLMAG-NWKMNLNHLEAIAHVQKLAFALADKDYDAVEVAVLAP     47
                       ....:|::.| :.||...|.:.:...:::| |||:: :.||.......
sco0578            1 MPAPAPSPVLLGVSLKMYFGHHQTLNWSRRIA-ALAER-HPAVTSGAARL     48

sco1945           48 FTDLRSVQTLVDGDKLKIKY----GAQDISAHDGGAYTGEISGPMLAKLK     93
                     |. |.:...||....:...|    |||||:..|.|.||||:.||:|.::.
sco0578           49 FV-LPTFPALVPATGILAPYGVALGAQDIATEDSGPYTGEVGGPVLKEIG     97

sco1945           94 CTYVAVGHSERRQYHAETDEIVNAKVKAAYKHGLTPILCVGEELDVREAG    143
                     |.|..|||:|||:.:.|.|.:|.||..||.::||||:|||||. |..:..
sco0578           98 CRYAEVGHAERRRLYGEGDTVVAAKTAAALRNGLTPVLCVGER-DAADPA    146

sco1945          144 NHVEHTLAQVE---GGLKDLAAEQAESVVIAYEPVWAIGTGKVCGADDAQ    190
                     :....|:|:.|   |||       :.:||:||||.||||..:...||...
sco0578          147 DAASRTVAEAERLLGGL-------SGAVVLAYEPQWAIGAPEPASADHIA    189

sco1945          191 EVCAAIRGKLAELYSQELADKVRIQYGGSVKSGNVAEIMAKPDIDGALVG    240
                     .||.|:||.| :.:.:.....|  .||||...|.:..:..  ..:|..:|
sco0578          190 TVCTALRGWL-DSHPRHAGSTV--IYGGSAGPGLLTRLAG--TAEGLFLG    234

sco1945          241 ---------GASLDSDEFVKIVRFRDQ------    258
                              ||.||        ..|..
sco0578          235 RFAHDPANVGAILD--------EIRSPAPAAVA    259
```

Supplementary Fig 6 Pairwise Sequence alignment paired between the protein sequences of *S. coelicolor* M145 triose phosphate isomerases (SCO1945, SCO0578). The alignment was performed with the EMBOSS Needle program.

```
SCO7040         MTVNDDSFTNWKHREETAESMIPMIGKLHREQDVTILLHSRSLVNKSVVSILKTHRFARQ 60
SCO1947         ------------------------------------------------------------ 0
SCO7511         ------------------------------------------------------------ 0

SCO7040         IAGAELSVTETMPFLRALTALDLGPSQIDIGMLAATYRTDDRGLSVEEFTAEAVAGATGA 120
SCO1947         ------------------------------------------------------------ 0
SCO7511         ------------------------------------------------------------ 0

SCO7040         NKIDRREGRDVVLYGFGRIGRLVARLLIEKAGSGNGLRLRAIVVRGGGEQDLVKRASLLR 180
SCO1947         ------MTIRVGINGFGRIGRNYFRALLEQGADIE-----IVAVND--LGDTATTAHLLK 47
SCO7511         -------MTRIGINGFGRIGRNVLRALLERDTKLE-----VVAVND--LTEPATLARLLA 46
                          : : *******   * *:*:  . :      :.*..    : .. * **

SCO7040         RDSVHGQFQGTITVDEDNSTILANGNAIKVIYADDPAQVDYTAYGIKDAILIDNTGKWRD 240
SCO1947         YDTILGRLKAEVSHTE--DTITVDGKTIKVLSERNPADIPWGELGV--DIVIESTGIFTK 103
SCO7511         FDSTAGRLGRPVTVDG--DTLVVDGHRIKVLAEREPARLPWAELGV--DIVLEATGRFTS 102
                 *:  *::   ::     .*: .:*: ***:   :** : :   *:   *::: ** : .

SCO7040         REGLSKHLRPGIDKVVLTAPGKGDVPNIVHGVNHDTIKPDE-RILSCASCTTNAIVPPLK 299
SCO1947         KADAEKHIAGGAKKVLISAPAKDEDITIVMGVNQDKYDPANHHVISNASCTTNCVAPMAK 163
SCO7511         ADAARAHLTAGARKVLVSAPSSGADVTLAYGVNTDAYDPAAHTIVSNASCTTNALAPLAA 162
                      *:  *  **:::**...   .:. *** *  .*    ::* ******.:.*

SCO7040         AMEDEYGVLRGHVETVHSFTNDQNLLDNYHKSDRRGRSAPLNMVITETGAASAVAKALPD 359
SCO1947         VLDENFGIVKGLMTTVHAYTNDQRILDFPHKDLRRARAAAENIIPTTTGAAKATALVLPQ 223
SCO7511         VLDELAGIEHGFMTTVHAYTQEQNLQDGPHRDARRARAAGVNIVPTTTGAAKAIGLVLPG 222
                .:::  *: :* : ***::*::*.: *  *:. **.*:*  *:: * ****.* . .**

SCO7040         LKARITGSSIRVPVPDVSIAILNLQLAREASREEVVDHLREVSLTSPLKRQIDFIGAPDA 419
SCO1947         LKGKLDGIAMRVPVPTGSATDLVVELQREVTKDEVNAAFKKAADDGDLKGILFYTE-DAI 282
SCO7511         LDGKLSGDSIRVPVPVGSIVELNTTVARDVTREDVLAAYRA-AADGPLAGVLEYSD-DPL 280
                *..:: * ::*****  * . *   : *:.::::*    :  :  . *   : :

SCO7040         VSSDFIGSRHASIVDAGALKV-EGDNAILYLWYDNEFGYSCQVVRVVQHVSGVEYPTYPA 478
SCO1947         VSSDITGDPASCTFDSSLTMVQEGKSVKILGWYDNEWGYSNRLVDLTVFVGNQL------ 336
SCO7511         VSSDITGNPASSIFDSALTRV-EGRHIKVVAWYDNEWGFSNRVIDTLALLATR------- 332
                ****: *.  :. .*:.   * **    :  *****:*:* :::     :.

SCO7040         PAV    481
SCO1947         ---    336
SCO7511         ---    332
```

Supplementary Fig 7 Multiple Sequence alignment paired between the protein sequences of *S. coelicolor* M145 glyceraldehyde 3-phopshate dehydrogenases (SCO7040, SCO1947, SCO7511). The alignment was performed using CLUSTAL O (1.2.4) Multiple sequence alignment.

```
sco4209                1 MADAPYKLILLRHGESEWNEKNLFTGWVDVNLTPKGEKEATRGGELLKDA     50
sco6818                1 --------------------------------------------------      0

sco4209               51 GLLPDVVHTSVQKRAIRTAQLALEAADRHWIPVHRHWRLNERHYGALQGK    100
sco6818                1 --------------------------------------------------      0

sco4209              101 DKAQTLAEFGEEQFMLWRRSYDTPPPALDRDAEYSQFSDPRYAMLPPELR    150
                                                             .|.|...:..|.:.
sco6818                1 ------------------------------------MSTPEPVLAGPGIL     14

sco4209              151 PQTECLKDVVGRMLPYWFDAIVPD---LLTGRT----VLVAAHGNSLR--    191
                                    .:|..|..|...|   |...||    .|||.|.::|.
sco6818               15 -----------LVLDGWGSADAADDNALSLARTPVLDELVAQHPSTLAEA     53

sco4209              192 --ALVKHLDG-ISDADIAGLNIPTGIPLSYELNAEFKPLNPGGTYLDPDA    238
                           ..|..|.| :.:::|..:.|..|.||.|:.....:.::.|.....|..
sco6818               54 SGEAVGLLPGTVGNSEIGHMVIGAGRPLPYDSLLVQQAIDSGALRSHPRL    103

sco4209              239 AAAAIEAVKNQGKKK-----------------------------------    253
                         .|...|.....|...
sco6818              104 DAVLNEVAATSGALHLIGLCSDGQIHAHVEHLSELLAAAATHQVERVFIH    153

sco4209              254 --------------------------------------------------    253

sco6818              154 AITDGRDVADHTGEAYLTRVAELAAAAGTGQIATVIGRGYAMDKAGDLDL    203

sco4209              254 --------------------------------------------------    253

sco6818              204 TERAVALVADGRGSPADSAHSAVHSSERGDEWVPASVLTEAGDARVADGD    253

sco4209              254 --------------------------------------------------    253

sco6818              254 AVLWFNFRSDRIQQFADRLHEHLTASGRTVNMVSLAQYDTRTAIPALVKR    303

sco4209              254 --------------------------------------------------    253

sco6818              304 ADASGGLADELQEAGLRSVRIAETEKFEHVTYYINGRDATVRDGEEHVRI    353

sco4209              254 --------------------------------------------------    253

sco6818              354 TGEGKADYVAHPHMNLDRVTDAVVEAAGRVDVDLVIANLANIDVVGHTGN    403

sco4209              254 --------------------------------------------------    253

sco6818              404 LAATVTACEATDAAVDQILQAARNSGRWVVAVGDHGNAERMTKQAPDGSV    453

sco4209              254 --------------------------------------------------    253

sco6818              454 RPYGGHTTNPVPLVIVPNRTDTPAPTLPGTATLADVAPTILHLLGHKPGP    503

sco4209              254 --------    253

sco6818              504 AMTGRPLL    511
```

Supplementary Fig 8 Pairwise Sequence alignment paired between the protein sequences of *S. coelicolor* M145 phosphoglycerate mutases (SCO4209, SCO6818). The alignment was performed with the EMBOSS Needle program.

```
Sco3096            1 --------MPSIDVVVAREILDSRGNPTVEVEVGLDDGSTGRAAVPSGAS     42
                             ..:|:.|.||.|:||||||||||:|.|:|||.||||||||||
Sco7638            1 MSATAVEPAAAIETVTARRIIDSRGNPTVEVDVVLEDGSLGRAAVPSGAS     50

Sco3096           43 TGAFEAIELRDGDPSRYLGKGVEKAVLAVIEQIGPELVGYDATEQRLIDQ     92
                     |||.||:||||.||:|:.||||::||..|..:|...:.|.||.:|..:|.
Sco7638           51 TGAREAVELRDEDPTRWHGKGVDRAVAHVNGEIAASVRGRDAADQAGLDA    100

Sco3096           93 AMFDLDATDNKGSLGANAILGVSLAVAHAASEASDLPLFRYLGGPNAHLL    142
                     |:..||.|..|..|||||:||||||.|.||:.|...||:|||||.:||||
Sco7638          101 ALIALDGTAAKSRLGANALLGVSLAAAKAAAAARRQPLYRYLGGADAHLL    150

Sco3096          143 PVPMMNILNGGSHADSNVDIQEFMIAPIGAESFSEALRWGAEVYHTLKKV    192
                     |:|||||:|||:|||:.:|.|||||.|:||::|:||:|.|:||:|||::.
Sco7638          151 PLPMMNIVNGGAHADNPLDFQEFMIVPVGADTFAEAVRMGSEVFHTLRRD    200

Sco3096          193 LKNKGLATGLGDEGGFAPNLGSNREALDLILEAIKEAGYTPGEQIALALD    242
                     |...|.:||:||||||||.|.:..||||.::.||:..||..|..|.|.:|
Sco7638          201 LLAAGHSTGVGDEGGFAPALRTAEEALDFVVAAIERTGYRAGPDIGLVMD    250

Sco3096          243 VAASEFYKDG--SYAFEGKNRSAAEMTEYYAELVEAYPLVSIEDPLFEDD    290
                     .|:|||::||  .||.||..||.||..::.|.|::|||:||||||:.|||
Sco7638          251 PASSEFFRDGGYDYAGEGVRRSPAEHADHLAGLIDAYPVVSIEDPMAEDD    300

Sco3096          291 WDGWNTITAKLGDKVQLVGDDLFVTNPERLARGIEENSANALLVKVNQIG    340
                     .|||..:|.::||:.||.|||:|.|:...:..||.....|::||||||||
Sco7638          301 LDGWRELTDRVGDRCQLTGDDVFCTDEALVREGIRTGVGNSVLVKVNQIG    350

Sco3096          341 SLTETLDAVELAQRNGFKCMMSHRSGETEDVTIADLAVATNCGQIKTGAP    390
                     :|||.|..|..|...|:..:||||||||||.|||||||||.|||||||:.
Sco7638          351 TLTEALATVATAHEAGWTVVMSHRSGETEDTTIADLAVATGCGQIKTGSL    400

Sco3096          391 ARSERVAKYNQLLRIEEILDDAAVYAGRSAFPRFKG    426
                     :||:|.||||:|:||||.|..:|.:|||||..|.
Sco7638          401 SRSDRTAKYNRLIRIEEELGASARFAGRSALRRV--    434
```

Supplementary Fig 9 Pairwise Sequence alignment paired between the protein sequences of *S. coelicolor* M145 enolases (SCO3096, SCO7638). The alignment was performed with the EMBOSS Needle program.

```
Sco2014              1 MRRAKIVCTLGPATDSYDQIKDLVDAGMDIARFNFSHGTHAEHEERYHRV     50
                         |||:|||||||||.||::|:..|::|||::|||||||||||||:.||.||
Sco5423              1 MRRSKIVCTLGPAVDSHEQLVTLIEAGMNVARFNFSHGTHAEHQGRYDRV     50

Sco2014             51 RKASDETGRSVGALADLQGPKIRLGHFGEGPVLLERGDSFTITVEEGVEG    100
                         |.|:.||||::|.|||||||||||..|.||||.|.|||.||||.|: |.|
Sco5423             51 RAAAKETGRAIGVLADLQGPKIRLETFAEGPVELVRGDEFTITAED-VPG     99

Sco2014            101 DRYICGTTYAGLAEDVTPGERVLVDDGKVCLEVTGVDGTRVRTRVIEGGM    150
                         ||.||||||.||..|||.|::||::||.|.|:||.|:|.||:|.|||||:
Sco5423            100 DRTICGTTYKGLPGDVTKGDQVLINDGNVELKVTEVEGPRVKTIVIEGGV    149

Sco2014            151 VSDHKGLNLPGVAVSVPALSKKDEDDLRWALRAGFDVVALSFVRSGRDIL    200
                         :|||||:||||.||:|||||:||.:|||:|||.|.|:|||||||..:|:.
Sco5423            150 ISDHKGINLPGAAVNVPALSEKDVEDLRFALRMGCDLVALSFVRDAKDVA    199

Sco2014            201 DVHRIMDEEGRRLPVIAKVEKPQAVENIEDIVAAFDGIMVARGDLGVEMP    250
                         ||||:|||||||:||||||||||||:|:||:|.||||:|||||||.||.|
Sco5423            200 DVHRVMDEEGRRVPVIAKVEKPQAVDNMEDVVMAFDGVMVARGDLAVEYP    249

Sco2014            251 LEQVPTVQKRAVKLAKRNAKPVIVATQMLDSMIDNARPTRAEASDVANAV    300
                         ||:||.||||.::|.:||||||||||||::|||.|:|||||||||||||:
Sco5423            250 LEKVPMVQKRLIELCRRNAKPVIVATQMMESMITNSRPTRAEASDVANAI    299

Sco2014            301 IDGTDAVMLSGETSVGKHPTDTVRTMARIVEAAEEDILAKGLPPLTERNK    350
                         :||.||||||.|:|||.:|.:||:||::||.|||:::|:|||.||....|
Sco5423            300 LDGADAVMLSAESSVGAYPIETVKTMSKIVTAAEQELLSKGLQPLVPGKK    349

Sco2014            351 PRTQGGAVARAAAEMGDFLGAKFLVAFTQSGDTVRRLSRYRSPIPLLAFT    400
                         ||||||:|||||||:.||||.|.||||||||||.|||||||:..|:||||
Sco5423            350 PRTQGGSVARAAAEIADFLGGKGLVAFTQSGDTARRLSRYRAAQPILAFT    399

Sco2014            401 PEPATRSQLSLTWGVETFLGPHADSTDAMVDQVDELLTRYGRCEKGDVVV    450
                         .:.:||:||:|:||||..:.|..::||.|||.||:...|.||...||.||
Sco5423            400 TDESTRNQLALSWGVEPHVVPFVNTTDEMVDLVDQETARLGRFSDGDTVV    449

Sco2014            451 ITAGSPPGVSGTTNLVRVHHIGEDDIPK     478
                         ||||||||||||||:|||.|:||....
Sco5423            450 ITAGSPPGVSGTTNMVRVLHLGETRRG-     476
```

Supplementary Fig 10 Pairwise Sequence alignment paired between the protein sequences of *S. coelicolor* M145 pyruvate kinases (SCO1214, SCO5423). The alignment was performed with the EMBOSS Needle program.
