# Supplementary material for: Transcriptional analysis of genes associated with glycolysis in Streptomyces coelicolor M145
Source: Int Microbiol. 2025 Nov 4;28(8):3053–63. doi: 10.1007/s10123-025-00744-6 (PMC12727718; doi:10.1007/s10123-025-00744-6)
Supplement: Supplementary file 2 — Supplementary Material 2(PDF 35.4 KB) [file 10123_2025_744_MOESM2_ESM.pdf]

Supplementary Table 1. Two-way ANOVA statistical analysis of gene expression

term	df	sumsq	meansq	statistic	p.value
GEN	31	248.511865	8.01651179	42.3576203	1.01E-69
tiempo	2	49.6371481	24.8185741	131.136305	1.24E-36
GEN:tiempo	62	140.956697	2.2734951	12.0126864	8.36E-41
Residuals	192	36.3375055	0.18925784	NA	NA
